# Investigation of the Relationship Between Health Literacy and Fall-Prevention Behaviours in Older Adults

**DOI:** 10.3390/healthcare14193302

**Published:** 2026-10-03

**Authors:** Nuray Girgin, Ayşe Göktaş, Mete Özgün

**Affiliations:** 1 Department of Physiotherapy and Rehabilitation, Faculty of Health Sciences, Ankara Medipol University, 06050 Ankara, Turkey; nuray.girgin@ankaramedipol.edu.tr; 2 Department of Occupational Therapy, Gulhane Faculty of Health Sciences, University of Health Sciences, 06018 Ankara, Turkey; ayse.goktas@sbu.edu.tr; 3 Department of Physiotherapy and Rehabilitation, Faculty of Health Sciences, Istanbul Health and Technology University, 34445 Istanbul, Turkey; 4 Institute of Biomedical Engineering, Bogazici University, 34684 Istanbul, Turkey

**Keywords:** fall, prevention, aged, health literacy, health behaviour, cross-sectional studies

## Abstract

**Background/Objectives:** Falls are a leading cause of morbidity and loss of independence in later life, and more than half are preventable. Health literacy has been proposed as a modifiable determinant of preventive behaviour, but the evidence rests largely on unadjusted associations and on outcomes such as fear of falling rather than on behaviour itself. This study examined how health literacy relates to fall-prevention behaviour in community-dwelling older adults, and whether that relationship holds once sociodemographic and health-related characteristics are considered. **Methods:** In this cross-sectional study, 253 adults aged 65 years and over completed a structured questionnaire comprising the Turkey Health Literacy Scale-32 (THLS-32) and the Falls Behavioural Scale for Older People (FaBS) between December 2023 and March 2024. The primary multivariable analysis used hierarchical multiple linear regression, with covariates selected on theoretical and clinical grounds and no automated variable selection. **Results:** Health literacy was inadequate or problematic–limited in 73.5% of participants. Health literacy and fall-prevention behaviour were negatively correlated (r = −0.304, *p* < 0.001). Exploratory subscale analyses showed the strongest inverse estimates for protective mobility (r = −0.388), practical strategies (r = −0.342) and avoidance (r = −0.331), whereas awareness-related subscales were not associated with health literacy. The sociodemographic and health-related covariate block explained 28.1% of the variance; in the final model, male sex, higher educational attainment, and assistive-device use were associated with FaBS scores. THLS-32 did not significantly improve the model (ΔR^2^ = 0.006, *p* = 0.151). **Conclusions:** Fall-prevention behaviour was more strongly associated with measured sociodemographic and health-related characteristics than with health literacy. Higher FaBS scores may partly reflect activity restriction as well as protective behaviour. These findings do not support the assumption that higher general health literacy alone is associated with greater use of the behaviours captured by the FaBS.

## 1. Introduction

Increases in life expectancy and the resulting growth of the older population have made the health and care needs of older adults an increasingly important public health concern [1]. Older adults often experience multiple age-related health problems that may limit their ability to perform activities of daily living, reduce their independence, and increase their reliance on others [1,2].

Falls are among the most common and serious health problems affecting older adults and are a leading cause of morbidity, mortality, and hospitalisation in this population [3,4]. Older adults who experience falls face an increased risk of death, hospitalisation, persistent injury, functional decline, and higher healthcare expenditure [5,6,7]. Falls among older adults have a multifactorial aetiology involving biological, behavioural, environmental, and socioeconomic factors. Age, sex, living alone, chronic diseases, medication use, and characteristics of the physical environment have all been associated with an increased risk of falling [8,9]. Behavioural and psychosocial factors including fear of falling, risk-taking or unsafe behaviours, improper use of assistive devices, inappropriate footwear, and physical inactivity may also contribute to fall risk [4,10].

Importantly, more than half of falls among older adults are considered preventable. Identifying individual and environmental risk factors and improving awareness of fall-prevention strategies are therefore essential [11,12,13]. Assessing fall-related risks encountered during activities of daily living may help older adults recognise potential hazards and adopt safer fall-prevention behaviours. Beyond their physical consequences, falls may cause anxiety and fear of falling, restrict daily activities, reduce independence, and negatively affect quality of life [2]. Early identification of age-related changes and modifiable fall-risk factors, together with the timely implementation of preventive interventions, may help preserve quality of life and reduce the burden on families and caregivers [14,15].

Health literacy may play an important role in supporting older adults’ health and well-being. It refers to an individual’s ability to access, understand, evaluate, and use health-related information and services to make informed health decisions [16]. It is therefore a set of cognitive and social competencies rather than a stock of factual knowledge, and the four competencies are not interchangeable: older adults often obtain health information without being able to appraise or apply it [17]. Health literacy includes competencies related to the safe use of medical devices and prescribed medicines, appropriate utilisation of healthcare services, and effective self-management [18,19]. Individuals with limited health literacy may experience difficulties accessing and navigating healthcare services and are more likely to have poorer health outcomes [20,21]. Evidence indicating that health literacy remains inadequate among substantial proportions of the population highlights the need for practical interventions to strengthen these competencies, particularly among older adults [22].

Although fall prevention and health literacy have each received considerable attention, evidence regarding the relationship between health literacy and fall-prevention behaviours among older adults remains limited. Three gaps stand out. First, most studies measure fear of falling, physical activity, or fall-related injury rather than the preventive behaviours themselves, so it is unclear whether health literacy translates into what older adults actually do [23]. Second, where behaviour itself has been examined [24], it has usually been summarised as a single score. Preventive behaviours are not homogeneous: some, such as scanning the floor for hazards, cost the person nothing, whereas others, such as avoiding ramps or crowded places, are achieved by giving up activity. Treating them as one construct may conceal opposing patterns, and instruments developed specifically to measure fall-related health literacy have so far been validated against knowledge rather than behaviour [25,26]. Third, a previous Turkish study linking health literacy to fall-prevention behaviour was conducted among hospitalised patients, whose behaviour is constrained by the ward environment [27]. The present study addresses these gaps in an online convenience sample by using a behaviour-specific instrument with ten subscales and examining associations at the level of individual subscales and separate health-literacy competencies. The following hypotheses were proposed: H1, fall-prevention behaviours are associated with the sociodemographic and health-related characteristics of older adults; and H2, health literacy is associated with fall-prevention behaviours over and above those characteristics.

## 2. Materials and Methods

### 2.1. Participants

**Figure 1:**
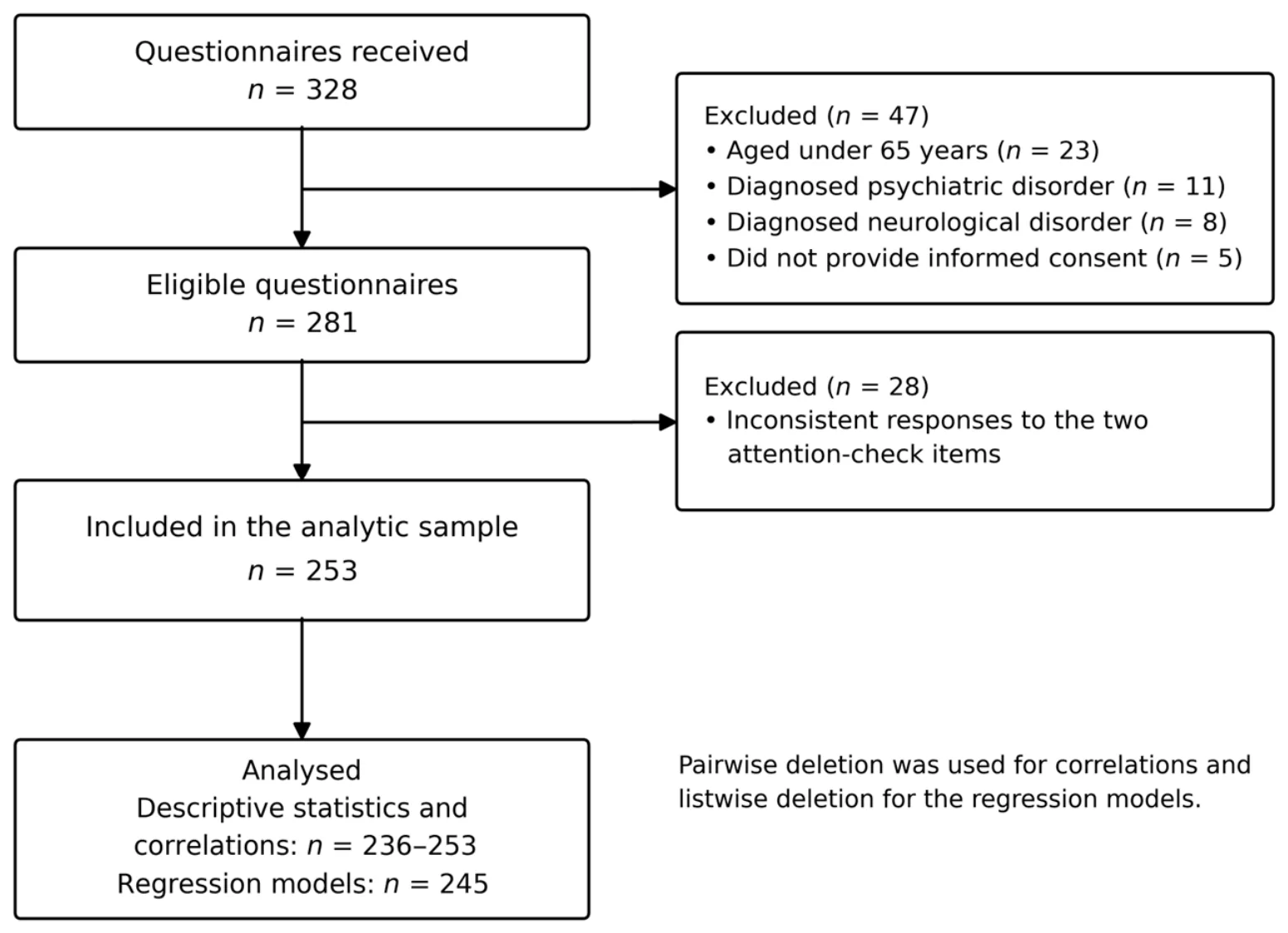
Flow of participants through the study. Item-level missing values within the analytic sample arose where participants indicated that an item did not apply to them or answered ‘no idea’ on the THLS-32.

This was an observational, cross-sectional study, reported in accordance with the STROBE statement for cross-sectional studies. The study was conducted between December 2023 and March 2024 using an online database (Google Forms). Participants were recruited by convenience sampling. The inclusion criteria were: (i) being aged 65 years or over; (ii) being able to read, write, and understand Turkish; (iii) being able to read and complete the questionnaire independently on a tablet or laptop computer; and (iv) providing informed consent. The exclusion criteria were: (i) complete hearing or vision loss; (ii) a diagnosed serious psychiatric or neurological disorder; (iii) refusal to participate or failure to provide informed consent; and (iv) failing the attention checks described below. Two attention-check items were embedded in the questionnaire. Each restated an earlier item in reversed form, so that inconsistent answers indicated inattentive responding or difficulty in following the content. Questionnaires with inconsistent answers to these items were excluded. A total of 328 questionnaires were received. Forty-seven were excluded at eligibility screening: 23 respondents were younger than 65 years, 11 reported a diagnosed psychiatric disorder, eight a diagnosed neurological disorder, and five did not provide informed consent. A further 28 questionnaires were excluded because of inconsistent responses to the two attention-check items, leaving 253 participants in the analytic sample (Figure 1). Informed consent forms were obtained from all participants.

### 2.2. Sample Size Calculation

The required sample size was estimated a priori using G*Power 3.1.9.7 [28]. The calculation was based on the association between health literacy and fall-prevention behaviour reported in a previous study of hospitalised older adults (r = −0.218) [27]. For a two-sided bivariate correlation test with α = 0.05 and 90% power, the minimum required sample size was 218. Recruitment exceeded this minimum to allow for exclusions, missing data, and incomplete responses. The final study sample comprised 253 participants, of whom 245 had complete data for the hierarchical multivariable regression analyses.

### 2.3. Study Protocol

Descriptive Information Form, Turkey Health Literacy Scale-32 (THLS-32) and Falls Behavioural Scale for Older People (FaBS) were used as data collection tools in the study.

### 2.4. Ethics Approval

Ethics approval for the study was obtained from the Istanbul Health and Technology University Ethics Board for Scientific, Social, and Non-Interventional Health Sciences Trials (Approval No: 2023/05-09, dated 30 October 2023). The study was conducted in accordance with the Declaration of Helsinki. All methods were performed in accordance with relevant guidelines and regulations. Before completing the questionnaire, all participants were informed about the purpose of the study, the anonymity of their responses, how their data would be stored and used, and their right to withdraw at any time without giving a reason.

### 2.5. Data Collection Instruments

#### 2.5.1. Descriptive Information Form

This form was designed to collect data on sex, age, level of education, cohabitants, presence of chronic illness, history of falls within the last year, hospitalisation due to falls, presence of healthcare workers at home, use of assistive devices, and overall health evaluation.

#### 2.5.2. Turkey Health Literacy Scale-32 (THLS-32)

The THLS-32 is a 32-item scale developed from the HLS-EU Working Conceptual Framework [29]. It comprises two dimensions, Treatment and Services and Disease Prevention and Health Promotion, each assessed through four competencies: Accessing, Understanding, Appraising, and Applying health information. The four scoreable response categories were coded as very easy = 4, easy = 3, difficult = 2 and very difficult = 1; responses of ‘no idea’ were treated as missing. For each scoreable index, the mean of valid item responses was transformed using (mean − 1) × 50/3, yielding a score from 0 to 50, with higher values indicating greater health literacy. A valid THLS-32 total score was available for 245 participants; eight participants did not have sufficient valid responses for a total index. The index is categorised as inadequate (0–25), problematic–limited (>25–33), sufficient (>33–42), or excellent (>42–50) health literacy. Okyay and Abacıgil reported a Cronbach’s alpha of 0.927 in the Turkish validation study [30]. In the present sample, internal consistency was α = 0.885 (McDonald’s ω = 0.886).

#### 2.5.3. Falls Behavioural Scale for Older People (FaBS)

The scale developed by Clemson et al. [31] in English consists of 30 items and 10 subscales. It assesses self-reported protective and precautionary behaviours and awareness related to potential falls in home life, lighting and vision, footwear, outdoor activities, and daily living. Each item is scored from 1 to 4. Items 7, 8, 9, 10, 19, and 23 were reverse-scored before analysis so that higher scores consistently represented greater use of the fall-related protective or precautionary behaviours represented by the scale. Subscale and total scores were calculated as item means and therefore range from 1 to 4. The subscales are Cognitive Adaptations (6 items), Protective Mobility (5 items), Avoidance (5 items), Awareness (4 items), Pace (2 items), Practical Strategies (3 items), Displacing Activities (1 item), Being Observant (1 item), Changes in Level (2 items), and Getting to the Phone (1 item). The scale has been validated for use in the Turkish older adult population [32]. In the present sample, internal consistency was α = 0.761 (McDonald’s ω = 0.803).

### 2.6. Statistical Analysis

The statistical analyses were conducted using jamovi v2.6. The Shapiro–Wilk test was applied to assess the normality of data distribution. Sample characteristics and scale scores are summarised as means with standard deviations, medians with interquartile ranges, and frequencies with percentages. Descriptive comparisons of scale scores across participant subgroups used Student’s *t*-test (effect size: Cohen’s d) or Welch’s one-way ANOVA (effect size: η^2^) with Games–Howell post-hoc tests, as appropriate. These comparisons were descriptive, did not inform variable selection and were not adjusted for multiple testing (Supplementary Table S1). As a secondary exploratory analysis, differences in FaBS total scores across the four THLS-32 categories were examined using the Kruskal–Wallis test, followed by Dunn’s pairwise tests with Holm adjustment. The primary total-score association between health literacy and fall-prevention behaviour was examined using Pearson correlation with a 95% confidence interval derived from Fisher’s z transformation. Correlations involving FaBS subscales and THLS-32 subscales or dimensions were exploratory; their *p* values were not adjusted for multiple testing and are interpreted as nominal. The primary multivariable analysis used hierarchical multiple linear regression with FaBS total score as the dependent variable. Covariates were selected on theoretical and clinical grounds from the study hypotheses and previous evidence. Two-sided *p* < 0.05 was used as the significance threshold for the primary analyses.

## 3. Results

### 3.1. Participant Characteristics

Of the 253 participants, 190 (75.1%) were women. Half of the sample (50.6%) was aged 65–69 years and 27 participants (10.7%) were 80 years or older. Most had attended primary or secondary school (55.7%), 49 (19.4%) held a higher education degree, and 63 (24.9%) were literate but had received no formal schooling. Chronic disease was reported by 148 participants (58.5%) and use of an assistive device by 45 (17.8%). A fall in the preceding 12 months was reported by 115 participants (45.5%); most of these had fallen once or twice, and 27 (23.5%) had been hospitalised as a result. Table 1 gives the full characteristics of the sample.

### 3.2. Health Literacy and Fall-Prevention Behaviour Scores

The mean THLS-32 total score was 27.6 ± 9.0 out of a possible 50. Scores were similar across the two dimensions and the four competencies of Accessing, Understanding, Appraising, and Applying health information (Table 2). Among the 245 participants with a valid THLS-32 total score, 85 (34.7%) had inadequate health literacy, 95 (38.8%) problematic–limited, 52 (21.2%) sufficient, and 13 (5.3%) excellent. Almost three-quarters of participants with a valid score (73.5%) therefore fell below the sufficient level.

The mean FaBS total score was 2.75 ± 0.50 on a scale ranging from 1 to 4. Cognitive adaptations and being observant were the behaviours participants reported most often, whereas protective mobility and practical strategies were reported least often.

**Figure 2:**
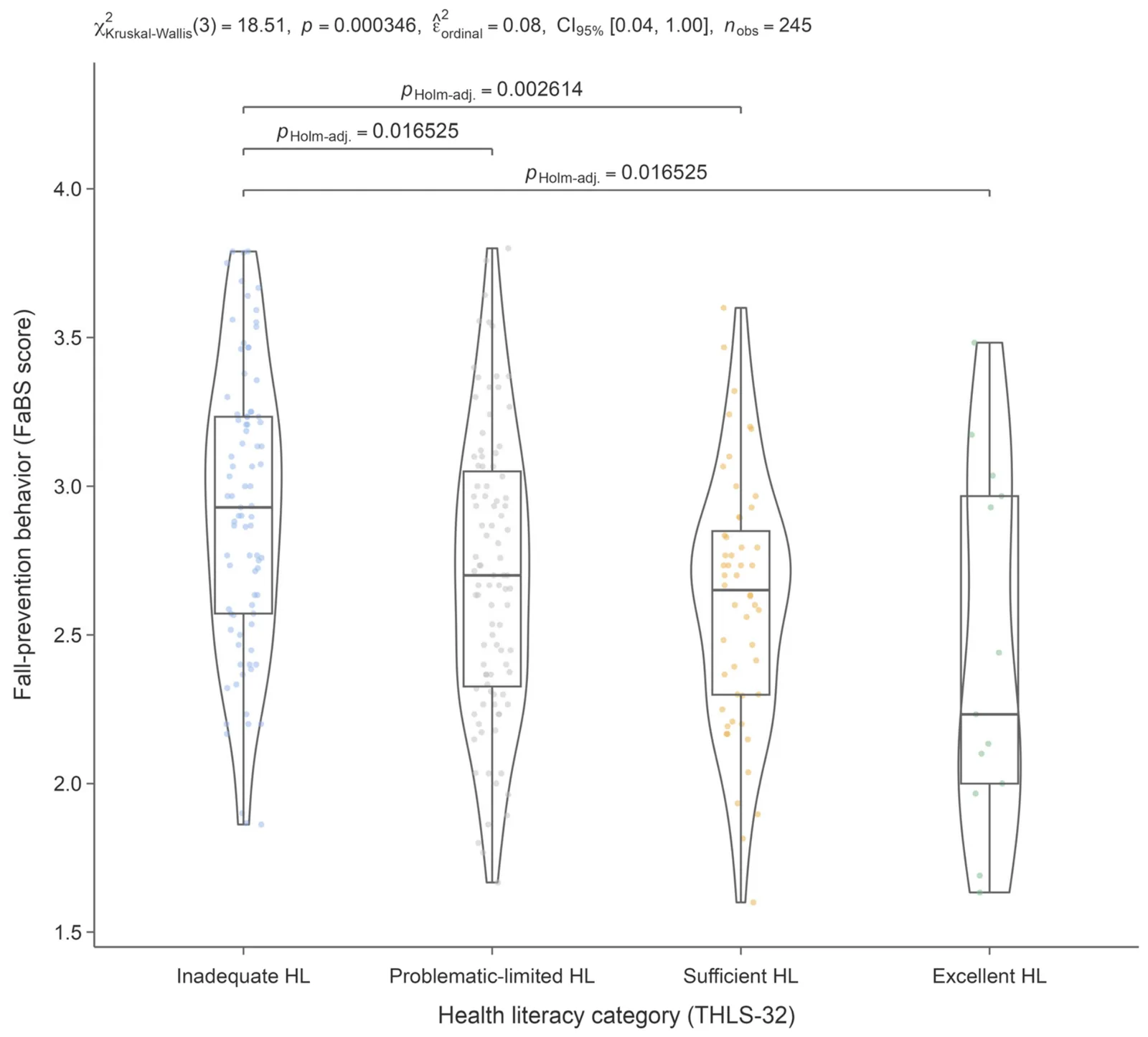
Distribution of FaBS total scores across THLS-32 health-literacy categories (*n* = 245). Boxes show medians and interquartile ranges, violins estimated densities, and points individual participants. Between-category differences were assessed using the Kruskal–Wallis test followed by Dunn’s pairwise tests with Holm adjustment; only statistically significant pairwise comparisons are shown. Higher FaBS scores indicate greater use of the behaviours represented by the scale.

As a secondary exploratory analysis, Figure 2 shows the distribution of FaBS total scores across the four health-literacy categories. Scores differed across categories (Kruskal–Wallis χ^2^(3) = 18.51, *p* < 0.001, ε^2^ = 0.08). Health literacy and fall-prevention behaviour scores across participant subgroups are presented in Supplementary Table S1.

### 3.3. Associations Between Health Literacy and Fall-Prevention Behaviour

**Figure 3:**
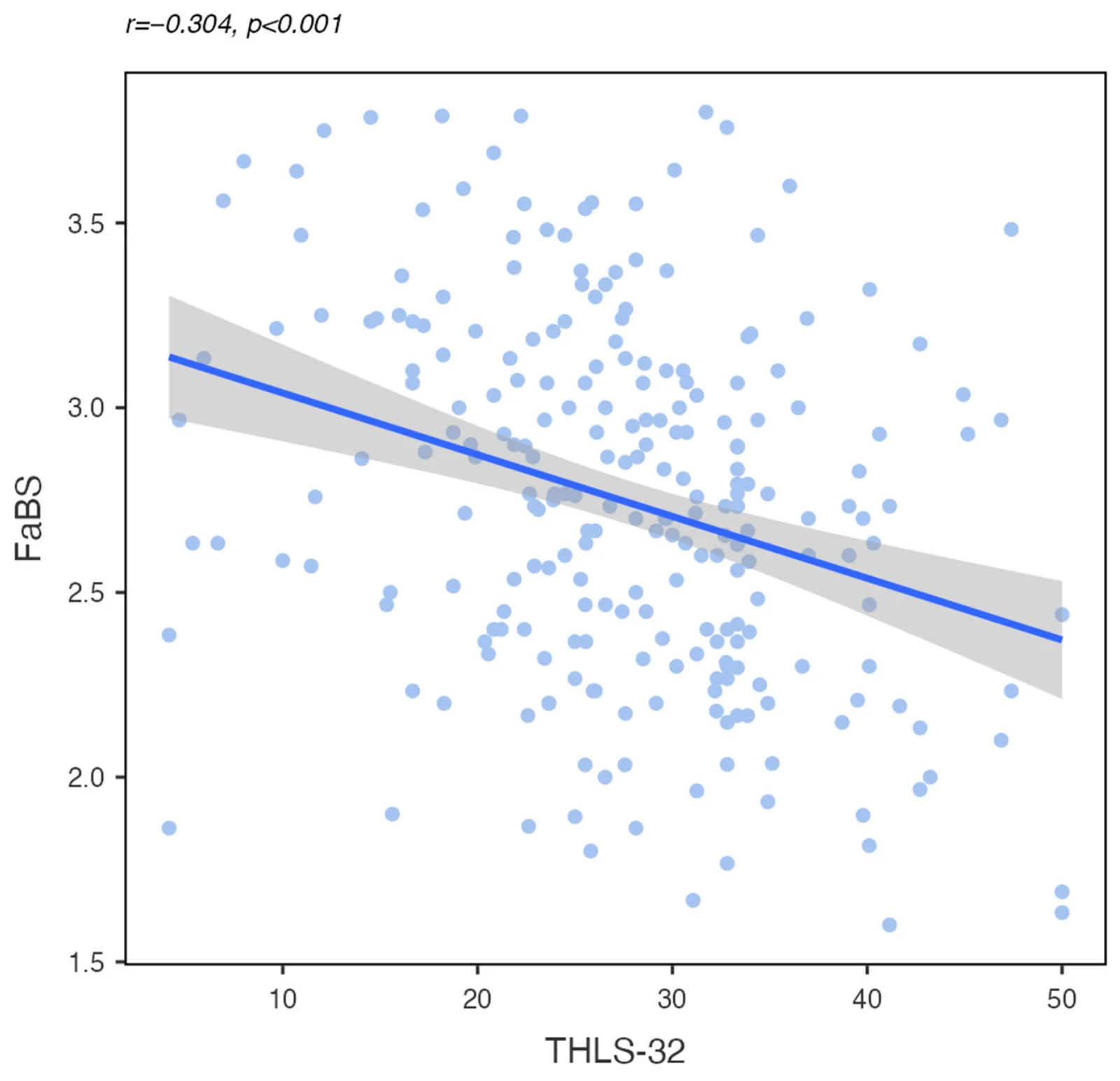
Association between health literacy (THLS-32 total score) and fall-prevention behaviour (FaBS total score); r = 0.304, *p* < 0.001, *n* = 245. The shaded band shows the 95% confidence interval. The association was attenuated and was no longer statistically significant after adjustment for sociodemographic and health-related characteristics (Table 4).

Health literacy and fall-prevention behaviour were negatively correlated (r = −0.304, 95% CI −0.414 to −0.186, *p* < 0.001; Figure 3). Participants with higher health literacy reported less frequent use of the behaviours measured by the FaBS.

**Figure 4:**
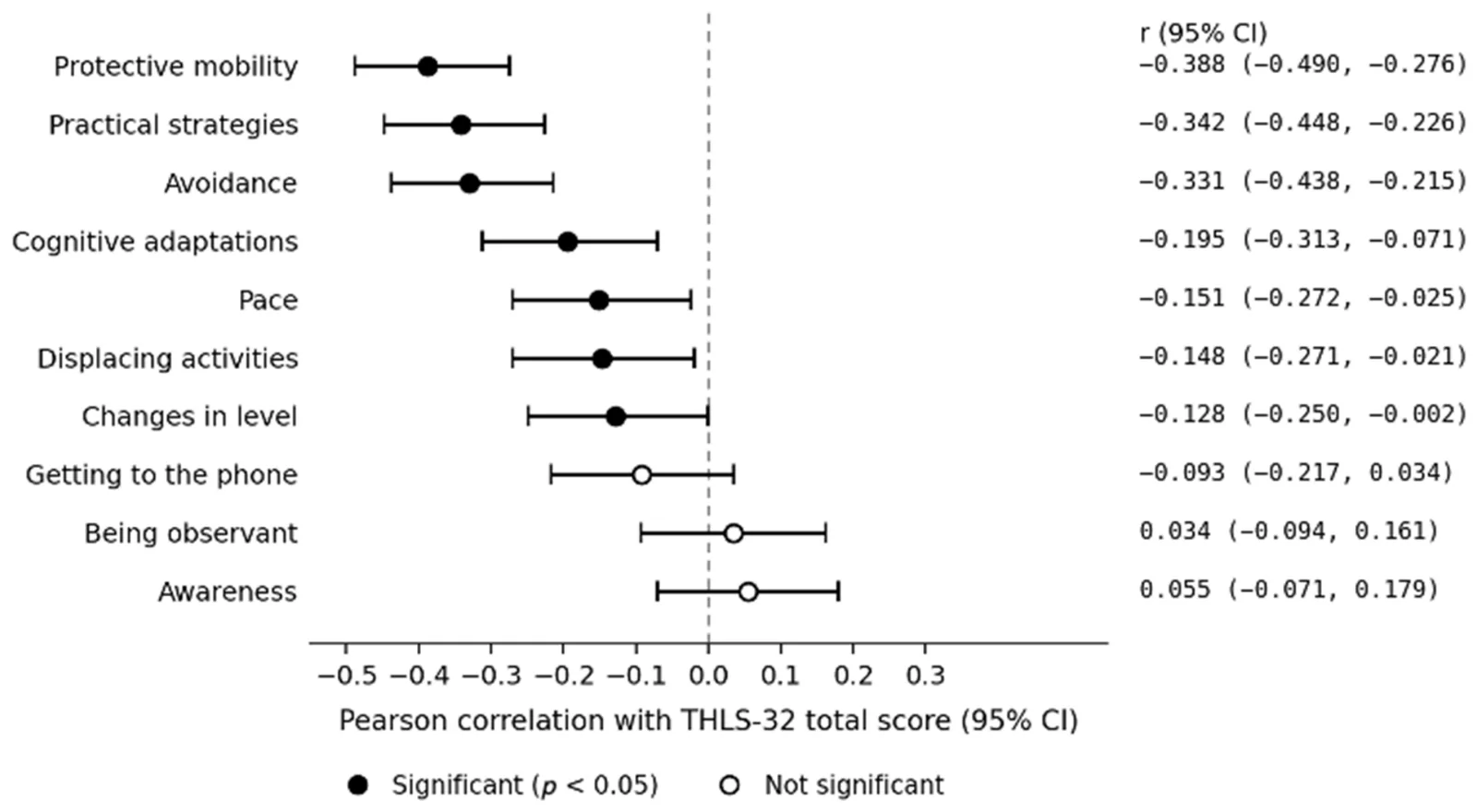
Exploratory Pearson correlations between health literacy (THLS-32 total score) and FaBS subscale scores. Points are correlation coefficients and horizontal lines are 95% confidence intervals derived from Fisher’s z transformation; subscales are ordered by effect size. Filled circles indicate nominal *p* < 0.05 and open circles nominal *p* ≥ 0.05; these *p* values were not adjusted for multiple testing. Higher FaBS scores indicate greater use of the behaviours represented by each subscale.

This association was not uniform across the FaBS subscales (Figure 4 and Table 3). Several subscales involving protective strategies or activity modification were negatively correlated with health literacy, most strongly protective mobility (r = −0.388), practical strategies (r = −0.342), and avoidance (r = −0.331). Two subscales that describe attention to hazards without any reduction in activity showed no statistically significant association: awareness (r = 0.055, *p* = 0.396) and being observant (r = 0.034, *p* = 0.608). Getting to the phone also showed no statistically significant association with health literacy (r = −0.093, *p* = 0.151).

All eight THLS-32 subscales were negatively correlated with the FaBS total score (Table 3). The correlation was stronger for the Treatment and Services dimension (r = −0.342) than for the Disease Prevention and Health Promotion dimension (r = −0.229).

**Table 3:** Correlations between health literacy and fall-prevention behaviour.

	r	95% CI	*p*
*FaBS subscales and total score with THLS-32 total score*			
Protective mobility	−0.388	−0.490, −0.276	<0.001
Practical strategies	−0.342	−0.448, −0.226	<0.001
Avoidance	−0.331	−0.438, −0.215	<0.001
Cognitive adaptations	−0.195	−0.313, −0.071	0.002
Pace	−0.151	−0.272, −0.025	0.019
Displacing activities	−0.148	−0.271, −0.021	0.023
Changes in level	−0.128	−0.250, −0.002	0.046
Getting to the phone	−0.093	−0.217, 0.034	0.151
Being observant	0.034	−0.094, 0.161	0.608
Awareness	0.055	−0.071, 0.179	0.396
FaBS total	−0.304	−0.414, −0.186	<0.001
*THLS-32 subscales, dimensions, and total score with FaBS total score*			
*Treatment and Services*			
Accessing	−0.287	−0.399, −0.166	<0.001
Understanding	−0.337	−0.444, −0.221	<0.001
Appraising	−0.238	−0.353, −0.116	<0.001
Applying	−0.313	−0.422, −0.195	<0.001
*Subtotal*	*−0.342*	*−0.448, −0.226*	*<0.001*
*Disease Prevention and Health Promotion*			
Accessing	−0.283	−0.396, −0.161	<0.001
Understanding	−0.223	−0.340, −0.100	<0.001
Appraising	−0.192	−0.312, −0.066	0.003
Applying	−0.154	−0.275, −0.028	0.016
*Subtotal*	*−0.229*	*−0.345, −0.106*	*<0.001*
THLS-32 total	−0.304	−0.414, −0.186	<0.001

Note. THLS-32 = Turkey Health Literacy Scale-32; FaBS = Falls Behavioural Scale for Older People; CI = confidence interval. Coefficients are Pearson product–moment correlations; confidence intervals were derived using Fisher’s z transformation. Within each THLS-32 dimension, accessing, understanding, appraising, and applying refer to the four competencies of the HLS-EU conceptual framework.

**Table 4:** Hierarchical multiple regression analysis predicting fall-prevention behaviour (FaBS total score; *n* = 245 for both models).

Variable	Model 1				Model 2			
	*B* (*SE*)	95% CI	β	*p*	*B* (*SE*)	95% CI	β	*p*
Constant	2.771 (0.408)	1.966, 3.575	—	<0.001	2.934 (0.423)	2.101, 3.767	—	<0.001
Sex: male	−0.174 (0.068)	−0.308, −0.039	−0.352	0.011	−0.173 (0.068)	−0.307, −0.039	−0.350	0.012
*Education (ref. literate)*								
Primary/secondary school	−0.142 (0.069)	−0.279, −0.005	−0.288	0.042	−0.115 (0.072)	−0.256, 0.026	−0.232	0.111
Higher education	−0.306 (0.097)	−0.496, −0.115	−0.619	0.002	−0.244 (0.105)	−0.452, −0.037	−0.494	0.021
*Living arrangement (ref. alone)*								
With spouse	−0.072 (0.082)	−0.235, 0.090	−0.146	0.381	−0.055 (0.083)	−0.219, 0.109	−0.112	0.508
Spouse and children	−0.131 (0.089)	−0.305, 0.044	−0.265	0.141	−0.120 (0.089)	−0.295, 0.054	−0.244	0.176
With children	0.122 (0.087)	−0.049, 0.293	0.247	0.161	0.128 (0.087)	−0.043, 0.299	0.259	0.141
Other	−0.149 (0.116)	−0.377, 0.079	−0.302	0.199	−0.139 (0.116)	−0.367, 0.089	−0.281	0.232
Chronic disease: yes	0.111 (0.060)	−0.008, 0.229	0.224	0.068	0.104 (0.060)	−0.015, 0.223	0.210	0.087
Fall history: yes	0.100 (0.059)	−0.016, 0.216	0.202	0.091	0.093 (0.059)	−0.023, 0.210	0.189	0.115
Healthcare worker at home: yes	−0.062 (0.066)	−0.192, 0.068	−0.125	0.350	−0.065 (0.066)	−0.195, 0.065	−0.131	0.328
Assistive device use: yes	0.244 (0.079)	0.089, 0.399	0.494	0.002	0.240 (0.078)	0.086, 0.395	0.486	0.002
Age (years)	0.001 (0.006)	−0.010, 0.012	0.008	0.897	<0.001 (0.006)	−0.011, 0.011	0.001	0.992
Health literacy (THLS-32)	—	—	—	—	−0.005 (0.004)	−0.012, 0.002	−0.096	0.151
*R^2^*	0.281				0.287			
*Adjusted R^2^*	0.243				0.247			
*F (df)*	7.54 (12, 232), *p* < 0.001				7.15 (13, 231), *p* < 0.001			
*ΔR^2^*	—				0.006			
*F for change in R^2^*	—				2.08, *p* = 0.151			

Note. FaBS = Falling Behaviour Scale for Older People; THLS-32 = Turkey Health Literacy Scale-32; CI = confidence interval; *B* = unstandardised coefficient; *SE* = standard error; β = standardised coefficient; ref. = reference category. Higher FaBS scores indicate more protective or restrictive fall-related behaviour. Binary predictors were coded with the category shown as 1 and its complement as 0. Predictors were entered simultaneously within each block and specified a priori on theoretical grounds; no automated variable selection was used. Block 1 comprised sociodemographic and health-related variables; the THLS-32 total score was added in Block 2. Multicollinearity was acceptable (VIF 1.01–1.19), residuals were independent (Durbin–Watson = 2.02) and homoscedastic (Breusch–Pagan *p* = 0.694), and no influential cases were identified (maximum Cook’s distance = 0.054).

### 3.4. Factors Associated with Fall-Prevention Behaviour

Block 1 of the hierarchical model explained 28.1% of the variance in FaBS total scores (adjusted R^2^ = 0.243; F(12, 232) = 7.54, *p* < 0.001). Four characteristics were independently associated with fall-prevention behaviour: sex (*p* = 0.011), educational attainment (*p* = 0.007), living arrangement (*p* = 0.039), and use of an assistive device (*p* = 0.002). Men scored 0.174 points lower than women. Participants with a higher education degree scored 0.306 points lower than those who were literate without formal schooling, and those who had attended primary or secondary school scored 0.142 points lower. Participants who used an assistive device scored 0.244 points higher than those who did not. Age was unrelated to fall-prevention behaviour (*p* = 0.897).

Adding health literacy in Block 2 did not significantly improve the model (ΔR^2^ = 0.006; F(1, 231) = 2.08, *p* = 0.151), and the THLS-32 total score was not independently associated with fall-prevention behaviour (B = −0.005, 95% CI −0.012 to 0.002, *p* = 0.151). The unadjusted association described above was therefore explained by the sociodemographic and health-related characteristics entered in Block 1.

Model assumptions were satisfied. Residuals were approximately normal and homoscedastic (Breusch–Pagan *p* = 0.694), independent (Durbin–Watson = 2.02), and no influential cases were identified (maximum Cook’s distance = 0.054). Variance inflation factors ranged from 1.01 to 1.19, indicating that collinearity among the predictors did not affect the estimates.

## 4. Discussion

This study examined how health literacy relates to fall-prevention behaviour in an online convenience sample of older adults. Three findings stand out. Health literacy was inadequate or problematic–limited in almost three-quarters of participants with a valid THLS-32 score. Health literacy and fall-prevention behaviour were inversely associated in the unadjusted analysis. After sociodemographic and health-related characteristics were included, however, health literacy did not explain statistically significant additional variance in behaviour.

Our study demonstrated that health literacy among older adults was markedly low. Based on the total health literacy score (THLS-32), 34.7% of participants were classified as having inadequate health literacy and a further 38.8% as problematic–limited, so that 73.5% fell below the sufficient level. Only 13 participants (5.3%) reached the excellent category. Scores were lower among participants with less education, those living with their children or alone, and those who rated their own health as poor. Consistent with these findings, Bakan and Yıldız reported in their study that 55.4% of participants had inadequate health literacy, 22.4% problematic–limited, 16.4% adequate, and 5.8% excellent [33].

Low health literacy is widespread in the general population, but it is consistently more pronounced in older age groups and among people with less education [29,34]. Our findings follow this pattern: almost three-quarters of participants scored below the sufficient level, and scores were lowest among those with no formal schooling. European population surveys have likewise reported that almost half of adults have limited health literacy, with older age and lower education associated with limited health literacy [29]. Kozak and Çevik Akyıl further showed that older adults may perform relatively well in accessing and evaluating health information yet struggle to understand and apply it in everyday decisions [35]. Information availability alone is therefore insufficient; the ability to translate knowledge into action matters just as much.

The high proportion of limited health literacy observed in our study, especially in older adults, underscores the urgent need for targeted interventions to strengthen health literacy not only in clinical settings but also in community and preventive health domains [27]. Addressing these gaps is essential for reducing health inequalities, improving quality of life in older populations, and alleviating the economic burden on healthcare systems. Poor communication between clinicians and patients with limited health literacy has, for example, long been described as a persistent source of avoidable cost and harm [36]. On a global level, the persistence of limited health literacy across all age groups highlights the importance of coordinated policies and awareness campaigns aimed at raising health literacy as a core component of public health strategies. At the same time, further research is required to develop and evaluate interventions that can improve health literacy in different populations.

The negative correlation we observed runs against the assumption that better health literacy leads to safer behaviour. The subscale analysis explains why. Health literacy was unrelated to the two subscales that describe watching for hazards (awareness and being observant) but was negatively related to every subscale that involves giving up or modifying activity, most strongly protective mobility, practical strategies, and avoidance. Exploratory correlations suggested differences in reported activity-modifying behaviours, but did not establish equivalent hazard awareness or differences in actual physical activity. Because the FaBS total score is driven mainly by the restriction subscales, the total inherits their direction. This is consistent with the closest previous Turkish study, which reported a negative correlation between total health literacy and FaBS scores among hospitalised older adults (r = −0.218) [27]. The original FaBS validation also linked higher behaviour scores with older age and lower mobility [31], indicating that more frequent protective or compensatory behaviour need not represent lower underlying fall vulnerability.

This distinction matters for interpretation of the FaBS. A high score is commonly described as protective, yet some items capture restriction or compensation. Avoiding slopes, crowds, or poor weather can reduce immediate exposure to hazards while also limiting physical activity, and inactivity is itself associated with fall risk [37]. Ellmers et al. similarly described concern about falling as potentially protective for some older adults and harmful for others [2]. Thus, FaBS scores may partly reflect activity restriction as well as informed self-protection; the present data do not quantify the relative contribution of these components.

Previous research links health literacy with medication management and other aspects of self-management in older adults [38]. In this sample, participants with chronic illness, assistive-device use, or poorer self-rated health tended to report fall-related behaviours more often. These characteristics may be markers of poorer health or possible functional limitation, but frailty and functional status were not measured directly and should not be inferred from these proxies.

All eight THLS-32 subscales correlated negatively with fall-prevention behaviour, but the association was consistently stronger for the Treatment and Services dimension than for the Disease Prevention and Health Promotion dimension. In other words, the aspect of health literacy that tracked behaviour most closely was the ability to deal with the healthcare system, not knowledge of preventive health. This is consistent with Kozak and Çevik Akyıl, who found that older adults manage to obtain health information but struggle to appraise and apply it [35], and it supports treating the competencies separately rather than as a single index.

In the adjusted model, four characteristics were independently associated with fall-prevention behaviour: sex, educational attainment, living arrangement and use of an assistive device. Women reported these behaviours more often than men, which matches Clemson et al.’s observation that women favour avoidance and cognitive adaptation strategies [31], and with reports of sex differences in the relationship between health literacy and falls [23]. Participants with less education reported them more often than university graduates, and participants who used a walking aid reported them more often than those who did not, a pattern also described among Turkish nursing-home residents [39]. Health literacy did not explain statistically significant additional variance in FaBS scores after adjustment. The association was attenuated after adjustment for the included sociodemographic and health-related characteristics. This is a useful caution: bivariate associations between health literacy and behaviour, which dominate this literature, may overstate what health literacy itself contributes.

A systematic review has emphasised that the most effective behavioural changes to reduce falls include exercise, walking, and balance training [40]. Comprehensive physical fitness programmes should therefore be recommended for fall prevention, and delivered in ways that are visually, auditorily, and cognitively accessible for older adults. Fall-prevention education has been shown to improve older adults’ awareness of fall-related hazards [41]. Our findings suggest, however, that awareness is not what distinguishes participants with higher and lower health literacy, and a recent scoping review has likewise noted how little behavioural evidence this field rests on [42].

Living arrangement was also associated with behaviour. Participants who lived with their children reported fall-related behaviours most often and those living with a spouse least often. Living with adult children may increase caution, either because the household is organised around the older person’s safety or because those who move in with their children are already more frail. Whichever explanation holds, the family context appears to shape day-to-day behaviour. Fall-prevention education is currently delivered mainly to older adults themselves or to health professionals [43,44], and extending it to family members may be worth evaluating.

Intervention studies are needed to evaluate the effects of programmes that strengthen older adults’ ability to understand and apply health information on fall-prevention behaviour [45]. In our sample, participants who rated their health as average or poor had lower health literacy and reported fall-related behaviours more often, which suggests that health perception, health literacy, and behaviour are closely intertwined in this population.

How health literacy relates to fall-related behaviour is still poorly understood, and the few available studies have reached different conclusions. A theoretical framework linking health literacy to concerns about falling has been proposed [46], but it has rarely been tested against observed behaviour. Chesser et al. reported fewer fall-related injuries among older adults with higher health literacy [47], whereas a meta-analysis found only a weak association between health literacy and physical activity [48]. Our results may offer one explanation for this inconsistency that the direction of the association depends on which behaviours are measured. Subscales that capture withdrawal from activity behave quite differently from those that capture attention to hazards and combining them into a single score may obscure this heterogeneity.

Cognitive factors significantly associated with physical activity in older adults include health literacy awareness and self-efficacy, and qualitative work suggests that older adults themselves see the ability to appraise and apply information as central to staying active [49]. Previous research suggests that age-related declines in cognitive functions increase the risk of limited health literacy, which can directly hinder the adoption of healthy behaviours and the effective management of chronic conditions [50,51]. Improving health literacy has elsewhere been linked to healthier lifestyles and greater engagement in physical activity [52], although we did not observe such an association once other characteristics were taken into account. A meta-analysis also reported that sedentary behaviour substantially increases fall risk in older adults [37]. Whether health literacy interventions can help older adults remain active therefore remains an open question.

Information and communication technologies offer one route for delivering tailored health content to older adults. Digital platforms, mobile health applications and telehealth services are being developed and evaluated as a means of providing continuous access to reliable information and interactive tools that support behaviour change, which is particularly relevant given the multiple health challenges, polypharmacy, and adherence difficulties common in this age group [53]. Whether such tools can influence fall-related behaviour, and in which direction, is a question for future work.

Taken together, these findings indicate that fall-prevention behaviour in this population is shaped more by sociodemographic position and health-related characteristics than by health literacy. Previous studies have examined falls and health literacy largely in isolation or have related health literacy to fear of falling rather than to behaviour. By using a behaviour-specific instrument and analysing its subscales separately, we were able to show that the association was strongest and most consistent for behaviours for activity modification or restriction. Fall-prevention programs should therefore be targeted to the groups identified here rather than delivered as general health literacy education and should be designed so that they do not encourage older adults to withdraw from activity [54].

This study has several strengths. The sample was community-based rather than drawn from an inpatient setting and was large enough to support a fully adjusted multivariable model with a comfortable margin. Since health literacy added nothing to the adjusted model, routinely recorded characteristics such as educational attainment, living arrangement, and use of a walking aid may offer a more practical starting point for identifying older adults who could benefit from fall-prevention support. Fall-prevention behaviour was measured with a validated, behaviour-specific instrument, and both health literacy and behaviour were examined at the level of their individual subscales rather than as single totals. The regression model was specified in advance on theoretical grounds, with no automated variable selection, and its assumptions were checked and reported in full.

### Limitations

Data were collected through an online platform which may have introduced selection bias by including only older adults who were able to use digital tools. In addition, a non-probability sampling technique, in which data are collected from participants who are most easily accessible, was used in this study. Although this approach allows rapid and economic data collection, it may limit the generalisability of the findings to the wider older adult population [55]. Several further limitations should be noted. The cross-sectional design means that no causal inference can be drawn, and the direction of the association between health literacy and behaviour cannot be established. Women made up three-quarters of the sample, which limits the precision of estimates for men. The descriptive subgroup comparisons involved many tests without correction for multiple testing and are reported for description only. Finally, internal consistency varied across the FaBS subscales and three of them consist of a single item, so the subscale-level findings on which part of our interpretation rests should be treated with caution.

## 5. Conclusions

In this sample of older adults, health literacy was inadequate or problematic–limited in almost three-quarters of participants with a valid THLS-32 score, but it did not explain statistically significant additional variance in fall-prevention behaviour after adjustment. Behaviour was explained instead by sex, educational attainment, living arrangement, and use of an assistive device. The association was inverse and was most evident for several behaviours involving activity modification or restriction. Accordingly, a high FaBS score may partly reflect compensatory caution or withdrawal as well as informed self-protection. Fall-prevention efforts should therefore be directed at the groups identified here and should be designed to preserve activity rather than to encourage older adults to do less. The results do not establish intervention effects or justify causal targeting of demographic groups. Longitudinal and interventional studies incorporating direct measures of mobility, frailty, fear of falling, and environmental risk are needed to determine whether improving health literacy changes fall-related behaviour while preserving safe activity.

### Practice Impact Statement

Fall-prevention programmes should not assume that general health-literacy education alone will increase the behaviours captured by the FaBS. Assessment and intervention planning should distinguish protective strategies that support safe activity from compensatory restriction or avoidance, and should consider mobility, functional vulnerability, and the individual’s home and social context. In community settings, educational attainment, living arrangement, and assistive-device use are readily available prompts for discussing communication needs, household support, and safe mobility. They should inform a broader assessment alongside fall history and functional evaluation.

## Figures and Tables

**Table 1:** Sociodemographic and health-related characteristics of the participants (*n* = 253).

Characteristic	*n* (%)
Sex	
Female	190 (75.1)
Male	63 (24.9)
Age group (years)	
65–69	128 (50.6)
70–74	65 (25.7)
75–79	33 (13.0)
≥80	27 (10.7)
Educational attainment	
Higher education	49 (19.4)
Primary/secondary school	141 (55.7)
Literate, no formal schooling	63 (24.9)
Living arrangement	
With spouse	71 (28.1)
With spouse and children	50 (19.8)
With children	58 (22.9)
Alone	48 (19.0)
Other	26 (10.3)
Health-related characteristics	
Chronic disease	148 (58.5)
Use of an assistive device	45 (17.8)
Healthcare worker in the household	57 (22.5)
Self-rated health	
Good	71 (28.1)
Average	162 (64.0)
Poor	20 (7.9)
Falls	
Fall in the preceding 12 months	115 (45.5)
Number of falls (of 115 fallers)	
1–2	97 (84.3)
3–5	14 (12.2)
≥6	4 (3.5)
Hospitalisation due to a fall (of 115 fallers)	27 (23.5)

Note. Percentages for the number of falls and for hospitalisation are calculated among the 115 participants who reported a fall in the preceding 12 months.

**Table 2:** Descriptive statistics and internal consistency of the THLS-32 and FaBS scales.

Scale, Dimension and Subscale	Mean ± SD	Median (Q1–Q3)	Possible Range	α/ω
Turkey Health Literacy Scale-32 (THLS-32)				
*Treatment and Services*				
Accessing	27.0 ± 12.5	29.2 (16.7–33.3)	0–50	
Understanding	28.0 ± 11.0	29.2 (20.8–33.3)	0–50	
Appraising	27.9 ± 10.1	29.2 (22.2–33.3)	0–50	
Applying	27.6 ± 10.9	29.2 (20.8–33.3)	0–50	
*Subtotal*	*27.6 ± 9.8*	*28.1 (22.2–33.3)*	*0–50*	
*Disease Prevention and Health Promotion*				
Accessing	27.3 ± 11.8	29.2 (20.8–33.3)	0–50	
Understanding	28.6 ± 10.3	29.2 (22.2–33.3)	0–50	
Appraising	27.8 ± 10.4	29.2 (20.8–33.3)	0–50	
Applying	27.5 ± 9.9	29.2 (20.8–33.3)	0–50	
*Subtotal*	*27.6 ± 9.1*	*29.2 (21.9–33.3)*	*0–50*	
THLS-32 total (32 items)	27.6 ± 9.0	28.0 (22.4–33.3)	0–50	0.885/0.886
Falls Behavioural Scale for Older People (FaBS)				
Cognitive adaptations (6 items)	3.05 ± 0.63	3.17 (2.67–3.50)	1–4	
Protective mobility (5 items)	2.34 ± 0.79	2.20 (1.80–2.80)	1–4	
Avoidance (5 items)	2.65 ± 0.67	2.67 (2.20–3.20)	1–4	
Awareness (4 items)	2.95 ± 0.67	3.00 (2.50–3.50)	1–4	
Practical strategies (3 items)	2.34 ± 0.68	2.33 (2.00–2.67)	1–4	
Pace (2 items)	2.93 ± 0.84	3.00 (2.50–3.50)	1–4	
Changes in level (2 items)	2.93 ± 0.67	3.00 (2.50–3.50)	1–4	
Displacing activities (1 item)	2.87 ± 0.90	3.00 (2.00–4.00)	1–4	
Being observant (1 item)	3.03 ± 0.98	3.00 (2.00–4.00)	1–4	
Getting to the phone (1 item)	2.68 ± 0.96	3.00 (2.00–3.00)	1–4	
FaBS total (30 items)	2.75 ± 0.50	2.73 (2.37–3.10)	1–4	0.761/0.803

Note. THLS-32 = Turkey Health Literacy Scale-32; FaBS = Falls Behavioural Scale for Older People; Q1–Q3 = 25th–75th percentiles; α = Cronbach’s alpha; ω = McDonald’s omega. Within each THLS-32 dimension the four subscales correspond to the competencies of accessing, understanding, appraising and applying health information.

## Data Availability

The data presented in this study are available on request from the corresponding author. The data are not publicly available because the ethical approval obtained for this study and the informed consent given by participants did not cover public deposition of individual-level data. Requests for access will be considered by the corresponding author and de-identified data can be shared with researchers who provide a methodologically sound proposal and appropriate ethical approval.

## Supplementary Materials

The following supporting information can be downloaded at: https://www.mdpi.com/article/10.3390/healthcare14193302/s1, Table S1: Health literacy and fall-prevention behaviour scores according to sociodemographic and health-related characteristics.

## Abbreviations

ANOVA	Analysis of variance
CI	Confidence interval
DPHP	Disease Prevention and Health Promotion
FaBS	Falls Behavioural Scale for Older People
HLS-EU	European Health Literacy Survey
SD	Standard deviation
STROBE	Strengthening the Reporting of Observational Studies in Epidemiology
THLS-32	Turkey Health Literacy Scale-32
TS	Treatment and Services
VIF	Variance inflation factor
