# Supplementary material for: Investigation of the Relationship Between Health Literacy and Fall-Prevention Behaviours in Older Adults
Source: Healthcare (Basel). 2026 Oct 3;14(19):3302. doi: 10.3390/healthcare14193302 (PMC13665292; doi:10.3390/healthcare14193302)
Supplement: Supplementary file 1 [file healthcare-14-03302-s001.zip › healthcare-4483756-supplementary.pdf]

Supplementary Materials

Supplementary Table S1. Health literacy and fall-prevention behaviour scores according to sociodemographic and health-related characteristics.

Characteristic	THLS-32 total	Treatment and services	Disease prevention and health promotion	FaBS total
Sex				
Female	27.23 ± 9.13	27.11 ± 9.91	27.27 ± 9.26	2.81 ± 0.49
Male	28.82 ± 8.46	29.04 ± 9.54	28.83 ± 8.51	2.57 ± 0.48
	t = −1.19, p = .236<br>d = −0.18	t = −1.32, p = .189<br>d = −0.20	t = −1.14, p = .256<br>d = −0.17	t = 3.38, p <.001<br>d = 0.49
Age group (years)				
65–69	29.78 ± 8.30^{a}	30.11 ± 8.89^{a}	29.30 ± 8.69^{a}	2.69 ± 0.47^{a}
70–74	25.82 ± 10.07^{b}	25.62 ± 10.79^{b}	26.05 ± 10.04^{a}	2.68 ± 0.50^{a}
75–79	25.25 ± 8.32^{b}	24.47 ± 9.97^{b}	26.20 ± 8.08^{a}	2.90 ± 0.52^{a}
≥80	24.98 ± 8.37^{ab}	24.63 ± 9.10^{b}	25.58 ± 9.14^{a}	2.93 ± 0.51^{a}
	F = 5.17, p = .003<br>η^2 = .059	F = 5.91, p = .001<br>η^2 = .068	F = 2.74, p = .049<br>η^2 = .033	F = 3.28, p = .025<br>η^2 = .042
Education				
Higher education	35.09 ± 7.74^{a}	35.70 ± 7.82^{a}	34.45 ± 8.38^{a}	2.44 ± 0.36^{c}
Primary/secondary	27.64 ± 7.93^{b}	27.74 ± 8.95^{b}	27.53 ± 7.95^{b}	2.74 ± 0.49^{b}
Literate, no formal schooling	21.55 ± 7.55^{c}	20.66 ± 7.98^{c}	22.42 ± 8.54^{c}	3.00 ± 0.46^{a}
	F = 42.2, p <.001<br>η^2 = .253	F = 49.1, p <.001<br>η^2 = .260	F = 27.4, p <.001<br>η^2 = .197	F = 25.7, p <.001<br>η^2 = .140
Living arrangement				
With spouse	29.16 ± 8.21^{ac}	29.86 ± 9.00^{a}	28.54 ± 8.09^{ab}	2.68 ± 0.42^{b}
With spouse and children	28.24 ± 8.88^{abc}	28.46 ± 9.02^{ab}	27.99 ± 9.81^{ab}	2.59 ± 0.39^{b}
With children	24.81 ± 8.62^{b}	24.22 ± 9.64^{b}	25.17 ± 8.66^{b}	3.03 ± 0.48^{a}
Alone	25.15 ± 9.60^{bc}	24.41 ± 10.78^{b}	25.98 ± 9.86^{b}	2.80 ± 0.55^{ab}
Other	33.07 ± 7.64^{a}	33.01 ± 8.14^{a}	33.05 ± 7.51^{a}	2.50 ± 0.53^{b}
	F = 5.92, p <.001<br>η^2 = .084	F = 6.46, p <.001<br>η^2 = .095	F = 4.75, p = .001<br>η^2 = .063	F = 8.66, p <.001<br>η^2 = .128
Chronic disease				
Yes	25.92 ± 9.41	25.91 ± 10.41	25.96 ± 9.42	2.86 ± 0.50
No	29.95 ± 7.82	29.87 ± 8.53	29.97 ± 8.12	2.60 ± 0.45
	t = −3.55, p <.001<br>d = −0.46	t = −3.16, p = .002<br>d = −0.41	t = −3.47, p <.001<br>d = −0.45	t = 4.26, p <.001<br>d = 0.54
Fall in the last year				
Yes	25.39 ± 8.46	25.28 ± 9.72	25.44 ± 8.47	2.86 ± 0.48
No	29.52 ± 9.01	29.54 ± 9.55	29.52 ± 9.22	2.65 ± 0.48
	t = −3.68, p <.001<br>d = −0.47	t = −3.45, p <.001<br>d = −0.44	t = −3.57, p <.001<br>d = −0.46	t = 3.47, p <.001<br>d = 0.44
Number of falls (fallers only)				
1–2	25.50 ± 8.21^{a}	25.29 ± 9.51^{a}	25.68 ± 8.28^{a}	2.86 ± 0.50^{a}
3–5	26.04 ± 9.31^{a}	26.64 ± 10.40^{a}	25.19 ± 9.26^{a}	2.82 ± 0.41^{a}
≥6	26.64 ± 6.02^{a}	26.49 ± 7.35^{a}	26.79 ± 5.09^{a}	3.19 ± 0.23^{a}
	F = 0.08, p = .928<br>η^2 = .001	F = 0.13, p = .878<br>η^2 = .003	F = 0.10, p = .904<br>η^2 = .001	F = 3.54, p = .075<br>η^2 = .017
Hospitalization due to a fall				
Yes	26.29 ± 7.58	26.69 ± 10.16	25.70 ± 6.55	2.98 ± 0.52
No	27.78 ± 9.12	27.61 ± 9.80	27.95 ± 9.39	2.74 ± 0.49
	t = 0.84, p = .404<br>d = 0.17	t = 0.47, p = .640<br>d = 0.09	t = 1.24, p = .216<br>d = 0.25	t = −2.50, p = .013<br>d = −0.50
Healthcare worker at home				
Yes	27.22 ± 9.19	27.95 ± 10.12	26.41 ± 9.48	2.68 ± 0.47
No	27.73 ± 8.94	27.46 ± 9.78	28.01 ± 8.97	2.77 ± 0.50
	t = −0.37, p = .712<br>d = −0.06	t = 0.33, p = .745<br>d = 0.05	t = −1.16, p = .248<br>d = −0.18	t = −1.18, p = .240<br>d = −0.18
Assistive device use				
Yes	24.91 ± 8.90	25.04 ± 10.24	25.09 ± 8.81	3.03 ± 0.55
No	28.19 ± 8.92	28.11 ± 9.70	28.18 ± 9.09	2.69 ± 0.46
	t = −2.19, p = .029<br>d = −0.37	t = −1.86, p = .064<br>d = −0.31	t = −2.01, p = .046<br>d = −0.34	t = 4.39, p <.001<br>d = 0.72
Self-rated health				
Good	31.99 ± 7.35^{a}	32.33 ± 7.59^{a}	31.65 ± 7.91^{a}	2.56 ± 0.43^{b}
Average	26.91 ± 8.56^{b}	26.78 ± 9.52^{b}	26.92 ± 8.71^{b}	2.80 ± 0.49^{a}
Poor	18.05 ± 8.62^{c}	17.34 ± 10.03^{c}	19.33 ± 9.18^{c}	3.05 ± 0.51^{a}
	F = 24.4, p <.001<br>η^2 = .164	F = 23.4, p <.001<br>η^2 = .159	F = 17.2, p <.001<br>η^2 = .128	F = 10.8, p <.001<br>η^2 = .077

Note. Values are mean ± SD. THLS-32 = Turkey Health Literacy Scale-32; FaBS = Falling Behaviour Scale for Older People. Higher THLS-32 scores indicate greater health literacy; higher FaBS scores indicate greater endorsement of fall-related behaviours. Two-group comparisons were made with independent-samples t-tests (effect size: Cohen's d); comparisons across three or more groups with Welch's one-way ANOVA (effect size: η^2) followed by Games-Howell post hoc test. Within each characteristic, group means in the same column that do not share a superscript letter differ significantly ($p < 0.05$); groups sharing a letter do not differ.
